# Prognostic Utility of the Combination of Platelet Count with Neutrophil-to-Lymphocyte Ratio in Aged Patients with Acute Myocardial Infarction Undergoing Percutaneous Coronary Intervention

**DOI:** 10.1155/2021/4023472

**Published:** 2021-04-23

**Authors:** Xiao-Qing Quan, Hong-Yan Ji, Jie Jiang, Jia-Bao Huang, Cun-Tai Zhang

**Affiliations:** ^1^Department of General Practice, Shenzhen Longhua District Central Hospital, The Affiliated Central Hospital of Shenzhen Longhua District, Guangdong Medical University, Shenzhen, China; ^2^Second Clinical Medical College, Tongji Medical College, Huazhong University of Science and Technology, Wuhan, China; ^3^Department of General Practice, The Third Affiliated Hospital of Sun Yat-sen University, Guangzhou, Guangdong, China; ^4^Department of Geriatrics, Tongji Hospital, Tongji Medical College, Huazhong University of Science and Technology, Wuhan, China

## Abstract

**Method:**

This was a study recording 637 patients who were diagnosed with acute myocardial infarction. Our patients were grouped according to the combination of platelet count and neutrophil-to-lymphocyte ratio. The prognostic role of the combination of platelet count and neutrophil-to-lymphocyte ratio on mortality was assessed by the univariate and multivariate Cox regression analysis.

**Result:**

Our study population was divided into three parts according to the median values of platelet count and neutrophil-to-lymphocyte ratio. It was indicated that platelet count and neutrophil-to-lymphocyte ratio were correlative mutually to a certain degree (*p*=0.010). The Kaplan–Meier analysis showed that the combination of high platelet count and high neutrophil-to-lymphocyte ratio had a greater risk of death in short- and long-term endpoints (log-rank *p*=0.046, *p* < 0.001, respectively). Moreover, by multivariate analysis, both high platelet count and high neutrophil-to-lymphocyte ratio groups were an independent predictor (hazard ratio: 2.132, 95% confidence interval: 1.020–4.454, *p*=0.044) and long-term mortality (hazard ratio: 2.791, 95% confidence interval: 1.406–5.538, *p*=0.003).

**Conclusion:**

The combination of platelet count and neutrophil-to-lymphocyte ratio could be a useful predictor for the prediction of in-hospital and long-term mortality in aged patients with acute myocardial infarction.

## 1. Introduction

Acute myocardial infarction (AMI) is the leading cause of cardiac death, especially for the aged people [1, 2]. Along with the prolongation of the life expectancy in the elderly population, cardiac death is more frequent in patients aged over 65 years old [3, 4]. The severe condition with insidious onset is one of the reasons for the increasing mortality in elderly AMI patients [3, 5, 6]. The current dilemma for clinical workers is figuring out a simple and powerful prognostic biomarker to identify high-risk patients.

Clinical and experimental evidence found that inflammation played a crucial role in the development and progression of AMI [7–9]. Previous studies showed that inflammatory markers, such as platelet count (PLC) and neutrophil-to-lymphocyte ratio (NLR), were connected with poor clinical outcomes in patients with AMI [10, 11]. Pathologically increased PLC in peripheral blood contributed to the initiation, progression, and complication of atherosclerosis [12–14]. According to a population-based cohort study, elevated PLC was associated with higher mortality and increased risk of cardiovascular events in AMI patients [15]. However, previous conflicting results showed that there was no correlation between elevated PLC and poorer cardiovascular endpoints [16–18].

Neutrophils and lymphocytes mirror the inflammatory status and have an effect on the destabilization of atherosclerotic plaque [19]. Accordingly, NLR has been assessed as a prognostic biomarker for cardiovascular diseases. A retrospective study by Han et al. showed that elevated NLR was a useful marker to predict long-term all-cause death in patients with AMI [20]. Nonetheless, the predictive value of NLR varies slightly in different studies, which is an imperfection for the risk stratification in AMI patients [21, 22].

Thus, to optimize the predictive value of PLC and NLR, some studies evaluated an original inflammation-related prognostic marker, named the combination of PLC and NLR (COP-NLR) [23, 24]. These studies showed that COP-NLR was a better prognostic predictor in patients with inflammatory diseases [23, 24]. However, to the best of our knowledge, the usefulness of COP-NLR has not been evaluated in AMI. Therefore, in the present study, we intended to investigate the prognostic value of COP-NLR in aged AMI patients.

## 2. Methods

### 2.1. Participants

This was a retrospective study which reviewed the data of 637 patients with AMI at the Tongji Hospital, Tongji Medical College, Huazhong University of Science and Technology, between January 2015 and October 2017. AMI was defined as either ST-segment elevation myocardial infarction (STEMI, typical symptoms of myocardial ischemia lasting for >30 min, with ST-segment elevation >1 mm in ≥2 contiguous standard or precordial leads and/or new onset of left bundle branch block) [25] and without ST-segment elevation myocardial infarction (NSTEMI, a rise of myocardial injury markers in combination with typical angina pectoris and without ST-segment elevation) [26]. The exclusion criteria were as follows: (a) all patients who were younger than 65 or older than 85 years; (b) patients with sepsis or trauma; (c) patients who were diagnosed with active cancer, autoimmune diseases, hematological proliferative diseases, chronic pulmonary disease, renal failure, and end-stage liver disease; and (d) patients who received steroid therapy or chemotherapy around the diagnosis index during six months.

This study has got approval by the ethics committee of Tongji Hospital, Tongji Medical College, Huazhong University of Science and Technology (TJ-C20141112), and is in accordance with the Helsinki Declaration.

### 2.2. Study Procedures and Laboratory Analysis

Venous blood samples were collected from all patients at the time of admission. Collection patients' demographic data were documented during hospitalization, including vital signs at admission, risk factors, medical history, and drug use. Laboratory results included creatinine, aminopherase, N-terminal prohormone of brain natriuretic peptide (NT-ProBNP), cardiac troponin I (CTnI), total cholesterol (TC), triglycerides, high-density lipoprotein cholesterol (HDL), and low-density lipoprotein cholesterol (LDL). The NLR was calculated as the ratio of the neutrophils to lymphocytes. Besides, echocardiographic parameters included left ventricular ejection fraction (LVEF).

Angiographic data including thrombolysis in myocardial infarction trial blood flow grade (TIMI) and Gensini score during the in-hospital period were obtained from the electronic medical records database. All included patients underwent coronary angiography. And the results of coronary angiography were determined by two professional cardiovascular doctors. Based on the results of coronary angiography and clinical findings, doctors selected using different treatment strategies according to the guideline [27]. In the perioperative period, anticoagulants, heparin/low molecular heparin, and tirofiban were used. For the patients with coronary stent placement, they were treated with aspirin and clopidogrel (or ticagrelor) as adjuvant antiplatelet therapy.

### 2.3. Study Endpoint and Follow-Up

The endpoints of our study were all-cause mortality that happened in-hospital and during the follow-up period. All-cause death was defined as mortality from any cause. The data during the hospitalization were obtained from the hospital administration system. And the information during follow-up was regularly collected from hospital records or the telephone interviews with patients or their relatives.

### 2.4. Statistical Analysis

In baseline characteristics analysis, continuous variables were presented as mean ± standard deviation and tested for normal distribution by the Kolmogorov–Smirnov test. The sample size of 224 patients was calculated to give a power of 90%, a type I error of 0.05, and a 10% drop-out rate. Comparative analyses between groups for continuous variables were performed by Student's *t*-test or Mann–Whitney *U* test. Categorical variables were summarized as percentages and compared with the *χ*^*2*^ test. Correlation between PLC and NLR was tested using the Spearman correlation coefficient. The mortality according to the median values of PLC and NLR were analyzed by the Kaplan–Meier method, and results were compared using the log-rank test. Univariate and multivariate analyses were performed to determine the significance of prognostic variables using the Cox proportional hazards model. Any variables examined in the univariate analysis for which the *p* value was <0.10 were contained in the multivariate model. A value of *p* < 0.05 was supposed to be statistically significant.

## 3. Results

### 3.1. Baseline Characteristics

**Figure 1:**
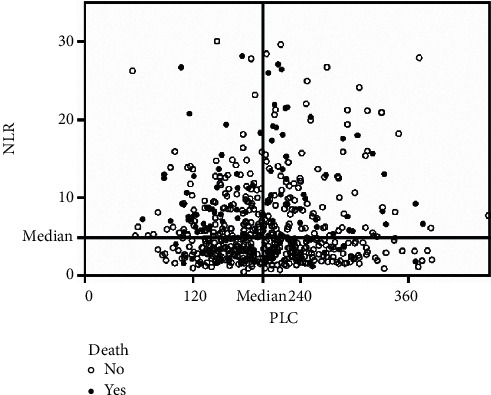
Correlation between PLC and NLR. When PLC cutoff value of 198.00 and NLR cutoff value of 4.88 were used, 34.04% mortality occurred in the high PLC and high NLR group (right upper quadrant). There was a weak but negative correlation between PLC and NLR: *r* = −0.102, *p*=0.010. Abbreviations: PLC, platelet count; NLR, neutrophil-to-lymphocyte ratio.

A total of 637 patients with AMI were enrolled in this study. A detailed description of demographic and laboratory characteristics is presented in Table 1. Median values of PLC and NLR were 198.00 and 4.88, respectively. There was a weak but negative correlation between PLC and NLR (Spearman *r* = −0.102, *p*=0.010; Figure 1). The patients were divided into 3 groups: low PLC (PLC < 198.00) and low NLR (NLR < 4.88) (*n* = 145) versus either low PLC or low NLR (*n* = 351) versus high PLC (PLC ≥ 198.00) and high NLR (NLR ≥ 4.88) (*n* = 141), as noted in Table 1. Both groups were predominantly male. The mean age of patients was 72.40 ± 5.28, 71.95 ± 5.20, and 72.31 ± 4.83 years, respectively, with an age range from 65 to 85 years. The high PLC and high NLR group had shorter reperfusion time (*p* < 0.001), longer hospitalization day, (*p*=0.024), and worse heart function (*p* < 0.001).

As shown in Table 2, in terms of the angiographic and procedural characteristics, no significant differences were discovered in the Gp IIbIIIa inhibitor use, stent use, TIMI grade, and Gensini score between the three groups, except for the culprit vessel quantity and the use of thrombus aspiration.

### 3.2. Clinical Outcomes

**Figure 2:**
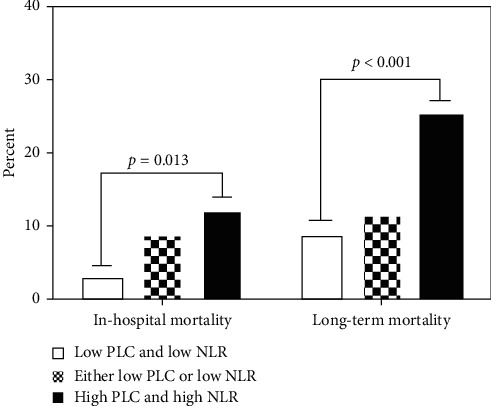
Clinical outcomes in 3 groups between in-hospital and long-term follow-up. Patients in the high PLC and high NLR group had significantly higher rates of all-cause mortality. Abbreviations: PLC, platelet count; NLR, neutrophil-to-lymphocyte ratio.

**Figure 3:**
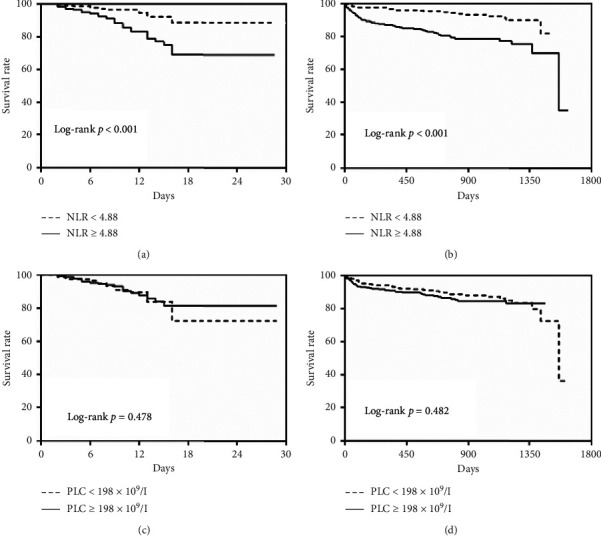
Kaplan–Meier survival curve of all-cause mortality based on the median value of NLR and PLC. (a) In-hospital mortality stratified by NLR. (b) Long-term mortality stratified by NLR. (c) In-hospital mortality stratified by PLC. (d) Long-term mortality stratified by PLC. Abbreviations: PLC, platelet count; NLR, neutrophil-to-lymphocyte ratio.

**Figure 4:**
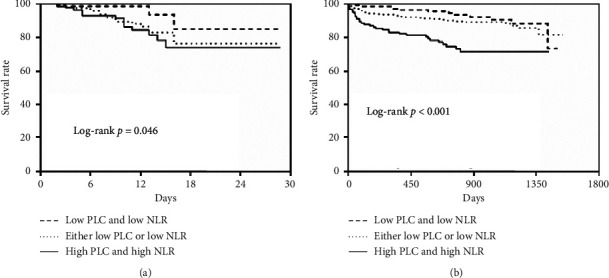
The all-cause mortality based on the combination of PLC and NLR. (a) Kaplan–Meier survival curve of in-hospital mortality. (b) Kaplan–Meier survival curve of long-term mortality. Abbreviations: PLC, platelet count; NLR, neutrophil-to-lymphocyte ratio.

During hospitalization and the average follow-up period was 771.71 ± 16.39 days, the all-cause mortality was significantly higher in patients with both high PLC and high NLR group (*p*=0.013 for in-hospital mortality, *p* < 0.001 for long-term mortality; Figure 2). The Kaplan–Meier curves based on the median values of NLR and PLC are shown in Figure 3. When PLC and NLR were compared between patients with and without death during short- and long-term follow-up, NLR was significantly higher in patients with death (both log-rank: *p* < 0.001; Figures 3(a) and 3(b)). However, there was no significant difference in PLC (log-rank: *p*=0.478 and *p*=0.482, resp.; Figures 3(c) and 3(d)). The Kaplan–Meier survival analysis according to the combination of PLC and NLR is shown in Figure 4. The mortality increased significantly in the high PLC and high NLR group during hospitalization (log-rank: *p*=0.046; Figure 4(a)) and long-term follow-up (log-rank: *p* < 0.001, Figure 4(b)).

### 3.3. Receiver Operating Characteristic Curve (ROC) Analysis

**Figure 5:**
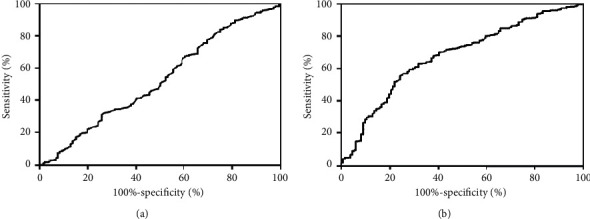
The ROC analysis of PLC and NLR. (a) ROC curves of PLC for mortality (*p*=0.361). (b) ROC curves of NLR for mortality (area under the curve = 0.677; 95% confidence interval: 0.626–0.728, *p* < 0.001). Abbreviations: ROC, receiver operating characteristic curve; PLC, platelet count; NLR, neutrophil-to-lymphocyte ratio.

As shown in Figure 5, the distinction does not prove a statistical significance in ROC analysis of PLC (*p*=0.361, Figure 5(a)). The area under the curve of the NLR with the outcomes of mortality was 0.677 (95% confidence interval [CI]: 0.626–0.728, *p* < 0.001; sensitivity = 75.00%, specificity = 56.40%; Figure 5(b)).

### 3.4. Independent Predictors of Mortality

In multivariate regression analysis, Killip class (hazard ratio [HR]: 1.810, 95% CI: 1.367–2.397, *p* < 0.001), LVEF (HR: 0.978, 95% CI: 0.957–0.999, *p*=0.040), history of coronary heart disease (CHD) (HR: 2.195, 95% CI: 1.130–4.264, *p*=0.020), neutrophil count (HR: 1.115, 95% CI: 1.044–1.190, *p*=0.001), HDL (HR: 0.311, 95% CI: 0.117–0.824, *p*=0.019), and the combination of high PLC and high NLR (HR: 2.132, 95% CI: 1.020–4.454, *p*=0.044) were found as independent predictors of in-hospital mortality (Table 3).

The combination of high PLC and high NLR was also found as an independent predictor of long-term mortality (HR: 2.791, 95% CI: 1.406–5.538, *p*=0.003) along with age (HR: 1.066, 95% CI: 1.020–1.115, *p*=0.005), Killip class (HR: 1.490, 95% CI: 1.165–1.905, *p*=0.001), LVEF (HR: 0.974, 95% CI: 0.956–0.992, *p*=0.005), and creatinine (HR: 1.006, 95% CI: 1.000–1.011, *p*=0.035) (Table 4).

## 4. Discussion

The inflammatory process has a central role in the development of atherosclerosis, instability of atherosclerotic plaques, and formation of thrombus [28–30]. The role of inflammatory markers on the prognosis of AMI has been suggested in previous studies [31, 32]. A large study of postmenopausal women showed that high PLC was associated with increased cardiovascular mortality [33]. Previous studies revealed that an elevated level of NLR was related to increased cardiovascular risk in patients with AMI [34, 35]. However, in the current study, PLC had no predictive significance in univariate or multivariate analysis. Some case-control studies and observational studies have also shown that there was no association between high PLC and risk of death [18, 36]. In the present study, NLR had a strong association with death in a patient with AMI. Yet, our results showed that NLR was not an independent predictor of short- and long-term mortality. Thus, further clinical studies are needed to explore a better biomarker to predict the death prognosis in AMI patients.

Recent studies found that the interaction of platelet and leukocyte is involved in the complex pathologic process of atherosclerosis [37]. Platelet–leukocyte interactions also activated cytokine expression, caused adhesion of endothelial cell, and promoted arterial thrombosis [38]. Based on this interaction of platelet with leukocyte, we made further research for the prognostic value of the combination of PLC and NLR in aged patients with AMI. The COP-NLR consisted of two biomarkers (PLC and NLR) related to cellular inflammation. It may reflect the balance of inflammation in the body more comprehensively, has stronger predictive potential for clinical outcomes, and contributes more to the risk stratification. COP-NLR has been identified as a novel predictive factor in patients with inflammatory diseases in previous studies [23, 24]. Our study showed that an increased level of COP-NLR was associated with a higher risk of in-hospital and long-term mortality in aged patients with AMI. Compared with individual PLC or NLR, the COP-NLR was a stronger predictor of mortality in aged AMI patients.

Although the pathologic mechanism between thrombocytosis and death prognosis has not been fully understood, several potential mechanisms have been described. Increased release of inflammatory mediators might lead to higher PLC [39]. The elevated platelet concentration represented greater possibilities to attach to the vessel wall, leading to platelet-dependent thrombus formation. In addition, on vulnerable coronary plaques, forming platelet-rich thrombi resulted in worse outcomes [40, 41].

The high NLR might probably be due to an increased neutrophil count and a decreased lymphocyte count. After the onset of AMI, increased cytokines, such as TNF-*α* and IL-6 caused relative neutrophilia [42]. Increased neutrophil counts predicted larger infarct size and adverse long-term cardiac prognosis [43]. Conversely, it was suggested that lymphocytopenia had a connection with AMI [44]. Similarly, Shiyovich and colleagues' study showed that there was a significant negative linear association between lymphocytes and death in the long-term follow-up period [45]. Lower lymphocyte counts might be owing to an abruptly increased level of corticosteroids and increased inflammation-related lymphocytes apoptosis [46, 47]. The NLR integrated for two cellular subtypes with opposite actions when it comes to vascular inflammation. According to Ayca's study, preprocedural NLR >4.9 was significantly related to stent thrombosis and higher mortality in patients with AMI [22]. However, the predictive value of NLR varied slightly in different studies [21, 22]. This was probably because of the differences in study design, population selection, statistical methods, outcome measurement.

It was indicated that LVEF and Killip class were important parameters for predicting adverse outcomes [48, 49]. Our study showed a similar result for predicting in-hospital and long-term prognosis of AMI. Along with COP-NLR, we also identified LVEF and Killip class as independent predictors of clinical outcomes in patients with AMI. Thus, this observation may support that the prognosis for patients with AMI is likely to be multifactorial. The identification of the potential predictor may provide a certain clinical assist for the risk stratification of AMI patients.

In the present study, our results showed that the COP-NLR was an independent predictor of all-cause mortality in AMI. This is the first report to investigate the value of combined PLC and NLR to predict the in-hospital and long-term mortality in aged AMI patients. Both PLC and NLR could be easily and widely available, calculated from WBC subtype counts, which were routinely performed at admission. Because of its easy obtainment and low cost, COP-NLR is possible for clinical application.

We have to acknowledge that there are some limitations in this study. Firstly, the retrospective design of the study sets a limit to the convincement of our study. Due to the nature of our study, the results must be explained with caution, given the possibility of confounders. Secondly, our endpoints are so simple that we have not investigated the relationship between COP-NLR with other major adverse cardiovascular events, including reinfarction, target vessel revascularization, arrhythmia, cerebrovascular accident, and congestive heart failure. Thirdly, we could not compare COP-NLR with other conventional inflammatory markers, for instance, C-reactive protein, fibrinogen, or myeloperoxidase. Because they were not routinely obtained in our study population.

## 5. Conclusion

In conclusion, a higher level of COP-NLR was independently associated with poor prognosis in aged patients with AMI. The COP-NLR was a potential predictor for short- and long-term mortality in aged AMI patients.

## Figures and Tables

**Table 1:** Baseline characteristics.

Characteristics	Low PLC and low NLR (*n* = 145)	Either low PLC or low NLR (*n* = 351)	High PLC and high NLR (*n* = 141)	*p*
Age, year	72.40 ± 5.28	71.95 ± 5.20	72.31 ± 4.83	0.615
Gender (male), *n* (%)	105 (72.41)	227 (64.67)	85 (60.28)	0.088
Smoke and drink, *n* (%)	59 (40.69)	140 (39.89)	54 (38.30)	0.914
Hypertension, *n* (%)	85 (58.62)	205 (58.40)	89 (63.12)	0.610
Prior CHD, *n* (%)	23 (15.86)	37 (10.54)	12 (8.51)	0.116
Diabetes, *n* (%)	35 (24.14)	93 (26.50)	38 (26.95)	0.831
Stroke, *n* (%)	15 (10.34)	54 (15.38)	16 (11.35)	0.237
SBP on admission (mmHg)	130.99 ± 24.30	130.01 ± 24.07	125.53 ± 25.94	0.117
DBP on admission (mmHg)	75.18 ± 14.22	75.61 ± 14.09	74.77 ± 16.14	0.806
HR on admission (beats/min)	73.43 ± 11.25	77.08 ± 15.59	82.74 ± 19.67	<0.001
Pain to reperfusion (h)	16.70 ± 8.14	14.16 ± 8.99	12.32 ± 8.27	<0.001
Hospitalization day	8.28 ± 5.12	8.44 ± 6.43	10.21 ± 9.50	0.024
Killip class, III-IV, *n* (%)	11 (7.59)	50 (14.25)	37 (26.24)	<0.001
*Diagnosis*				
STEMI	57 (39.31)	209 (59.54)	95 (67.38)	<0.001
NSTEMI	88 (60.69)	142 (40.46)	46 (32.62)	<0.001
LVEF (%)	55.86 ± 12.33	52.72 ± 12.21	49.92 ± 12.27	<0.001
Creatinine (*μ*mol/l)	87.10 ± 26.91	92.98 ± 39.34	93.60 ± 35.44	0.202
AST (u/l)	55.45 ± 68.72	90.26 ± 106.13	136.06 ± 129.10	<0.001
ALT (u/l)	28.68 ± 39.30	36.15 ± 37.78	43.52 ± 44.94	0.007
NT-proBNP (pg/ml)	3863.64 ± 6575.65	4813.60 ± 7299.85	5702.91 ± 11031.69	0.161
CTnI (pg/ml)	10058.40 ± 15443.85	14747.61 ± 18692.72	22783.39 ± 20504.55	<0.001
HDL (mmol/l)	1.09 ± 0.41	1.08 ± 0.44	1.17 ± 0.50	0.124
LDL (mmol/l)	2.52 ± 0.92	2.52 ± 0.92	2.79 ± 1.09	0.012
Total triglyceride (mmol/l)	1.36 ± 0.81	1.39 ± 1.09	1.20 ± 1.08	0.169
Total cholesterol (mmol/l)	4.00 ± 1.07	4.04 ± 1.09	4.42 ± 1.25	0.001
WBC count (10^9^/l)	6.97 ± 2.35	9.00 ± 3.19	12.37 ± 4.58	<0.001
PLC (10^9^/l)	153.80 ± 26.76	201.10 ± 65.48	258.08 ± 62.10	<0.001
Neutrophil count (10^9^/l)	4.62 ± 1.86	6.97 ± 3.32	10.70 ± 4.29	<0.001
Lymphocyte count (10^9^/l)	1.67 ± 0.63	1.42 ± 0.68	1.02 ± 0.47	<0.001
Monocyte count (10^9^/l)	0.52 ± 0.20	0.56 ± 0.28	0.61 ± 0.39	0.025
Hemoglobin (mg/dl)	129.19 ± 18.56	126.94 ± 19.25	123.43 ± 17.86	0.033
NLR	2.92 ± 1.03	6.61 ± 6.35	12.29 ± 7.17	<0.001
*Medications in hospital, n (%)*				
Aspirin	140 (96.55)	338 (96.30)	132 (93.62)	0.356
Clopidogrel	135 (93.10)	337 (96.01)	131 (92.91)	0.244
Beta-blocker	108 (74.48)	250 (71.23)	94 (66.67)	0.342
ACEI/ARB	109 (75.17)	244 (69.52)	92 (65.25)	0.184
Statin	144 (99.31)	343 (97.72)	136 (96.45)	0.254

Mean ± SD and *n* (%) are reported for continuous and categorical variables, respectively. Abbreviations: SD, standard deviation; PLC, platelet count; NLR, neutrophil-to-lymphocyte ratio; COP-NLR, combination of platelet count and neutrophil-to-lymphocyte ratio; CHD, coronary heart disease; SBP, systolic blood pressure; DBP, diastolic blood pressure; HR, heart rate; STEMI, ST-segment elevation myocardial infarction; NSTEMI, non-ST-elevation myocardial infarction; LVEF, left ventricular ejection fraction; AST, aspartate aminotransferase; ALT, alanine aminotransferase; NT-proBNP, N-terminal probrain natriuretic peptide; CTnI, cardiac troponin I; HDL, high-density lipoprotein cholesterol; LDL, low-density lipoprotein cholesterol; WBC, white blood cell; SII, systemic immune-inflammatory index; ACEI/ARB, angiotensin-converting enzyme inhibitor/angiotensin II receptor blocker.

**Table 2:** Angiographic and procedural characteristics in the study population.

Characteristics	Low PLC and low NLR (*n* = 145)	Either low PLC or low NLR (*n* = 351)	High PLC and high NLR (*n* = 141)	*p*
*Culprit vessel, n (%)*				0.102
LAD	130 (89.66)	305 (86.89)	123 (87.23)	
LCX	98 (67.59)	225 (64.10)	85 (60.28)	
RCA	103 (71.03)	216 (61.54)	92 (65.25)	

*Number of diseased vessels, n (%)*				<0.001
0	1 (0.69)	0 (0.00)	0 (0.00)	
1	27 (18.62)	101 (28.77)	43 (30.50)	
2	47 (32.41)	105 (29.91)	37 (26.24)	
3	70 (48.28)	145 (41.31)	61 (43.26)	
Use of thrombus aspiration, *n* (%)	7 (4.83)	34 (9.69)	27 (19.15)	<0.001
Gp IIbIIIa inhibitor use, *n* (%)	124 (85.52)	293 (83.48)	112 (79.43)	0.371
Stent use, *n* (%)	114 (78.62)	264 (75.21)	98 (69.50)	0.197

*Preprocedural TIMI grade, n (%)*				1.000
0	73 (50.34)	223 (63.53)	98 (69.50)	
1	65 (44.83)	116 (33.05)	38 (26.95)	
2	6 (4.14)	10 (2.85)	5 (3.55)	
3	1 (0.69)	2 (0.57)	0 (0.00)	

*Postprocedural TIMI grade, n (%)*				1.000
0	10 (6.90)	50 (14.25)	12 (8.51)	
1	9 (6.21)	18 (5.13)	21 (14.89)	
2	5 (3.45)	16 (4.56)	4 (2.84)	
3	121 (83.45)	267 (76.07)	104 (73.76)	
Gensini score (SD)	98.86 (60.95)	89.62 (46.64)	98.26 (57.20)	0.103

Mean (SD) and *n* (%) are reported for continuous and categorical variables, respectively. Abbreviations: SD, standard deviation; PLC, platelet count; NLR, neutrophil-to-lymphocyte ratio; COP-NLR, combination of platelet count and neutrophil-to-lymphocyte ratio; LAD, left coronary artery; LCX, left circumflex; RCA, right coronary artery; TIMI, thrombolysis in myocardial infarction.

**Table 3:** Predictors of in-hospital mortality in univariable and multivariable Cox regression analyses.

Variables	Univariable			Multivariable		
	HR	95% CI	*p*	HR	95% CI	*p*
Age	1.026	0.975–1.079	0.331			
Gender (male)	1.457	0.838–2.533	0.182			
Gensini score	1.007	1.002–1.011	0.005	NS	NS	NS
Killip class	2.282	1.779–2.927	<0.001	1.810	1.367–2.397	<0.001
LVEF	0.954	0.935–0.974	<0.001	0.978	0.957–0.999	0.040
Smoke and drink	1.521	0.832–2.779	0.173			
Hypertension	1.075	0.615–1.878	0.800			
Prior CHD	2.274	1.192–4.338	0.013	2.195	1.130–4.264	0.020
Diabetes	1.106	0.618–1.979	0.734			
Total cholesterol	0.834	0.649–1.070	0.153			
Total triglyceride	0.932	0.691–1.255	0.641			
HDL	0.318	0.115–0.877	0.027	0.311	0.117–0.824	0.019
LDL	0.918	0.691–1.220	0.554			
Creatinine	1.010	1.005–1.016	<0.001	NS	NS	NS
WBC count	1.119	1.062–1.180	<0.001	NS	NS	NS
Neutrophil count	1.144	1.087–1.205	<0.001	1.115	1.044–1.190	0.001
Lymphocyte count	0.839	0.545–1.293	0.426			
PLC > 198.00	1.220	0.702–2.119	0.481			
NLR > 4.88	1.045	1.019–1.072	0.001	NS	NS	NS
PLC > 198.00 and NLR > 4.88	3.476	1.164–10.383	0.026	2.132	1.020–4.454	0.044

HR, hazard ratio; CI, confidence interval; NS, no statistical significance; CHD, coronary heart disease; LVEF, left ventricular ejection fraction; HDL, high-density lipoprotein cholesterol; LDL, low-density lipoprotein cholesterol; WBC, white blood cell; PLC, platelet count; NLR, neutrophil-to-lymphocyte ratio.

**Table 4:** Predictors of long-term mortality in univariable and multivariable cox regression analyses.

Variables	Univariable			Multivariable		
	HR	95% CI	*p*	HR	95% CI	*p*
Age	1.082	1.039–1.126	<0.001	1.066	1.020–1.115	0.005
Gender (male)	1.487	0.954–2.317	0.080	NS	NS	NS
Gensini score	1.002	0.998–1.006	0.225			
Killip class	2.011	1.009–2.513	<0.001	1.490	1.165–1.905	0.001
LVEF	0.960	0.944–0.977	<0.001	0.974	0.956–0.992	0.005
Smoke and drink	1.110	0.705–1.747	0.652			
Hypertension	1.262	0.797–1.998	0.321			
Prior CAD	1.010	0.486–2.098	0.978			
Diabetes	1.372	0.848–2.218	0.197			
Total cholesterol	1.035	0.850–1.261	0.729			
Total triglyceride	0.992	0.784–1.255	0.948			
HDL	1.338	0.870–2.057	0.185			
LDL	1.037	0.825–1.303	0.756			
Creatinine	1.009	1.005–1.014	<0.001	1.006	1.000–1.011	0.035
WBC count	1.111	1.058–1.167	<0.001	NS	NS	NS
Neutrophil count	1.118	1.066–1.173	<0.001	NS	NS	NS
Lymphocyte count	0.564	0.376–0.847	0.006	NS	NS	NS
PLC > 198.00	1.001	0.998–0.004	0.549			
NLR > 4.88	1.051	1.028–1.074	<0.001	NS	NS	NS
PLC > 198.00 and NLR > 4.88	3.695	1.893–7.212	<0.001	2.791	1.406–5.538	0.003

HR, hazard ratio; CI, confidence interval; NS, no statistical significance; CHD, coronary heart disease; LVEF, left ventricular ejection fraction; HDL, high-density lipoprotein cholesterol; LDL, low-density lipoprotein cholesterol; WBC, white blood cell; PLC, platelet count; NLR, neutrophil-to-lymphocyte ratio.

## Data Availability

The data used to support the findings of this study are available from the corresponding author upon request.
